# Drivers of melioidosis endemicity: epidemiological transition, zoonosis, and climate change

**DOI:** 10.1097/QCO.0000000000000827

**Published:** 2022-04-28

**Authors:** Emma Birnie, Jason J. Biemond, W. Joost Wiersinga

**Affiliations:** a Amsterdam UMC location University of Amsterdam, Center for Experimental and Molecular Medicine; b Amsterdam UMC location University of Amsterdam, Division of Infectious Diseases, Meibergdreef 9, Amsterdam, Netherlands

**Keywords:** *Burkholderia pseudomallei*, climate change, epidemiological transition, melioidosis, one health, zoonosis

## Abstract

Melioidosis, caused by the soil-dwelling bacterium *Burkholderia pseudomallei*, is a tropical infection associated with high morbidity and mortality. This review summarizes current insights into melioidosis’ endemicity, focusing on epidemiological transitions, zoonosis, and climate change.

**Recent findings:**

Estimates of the global burden of melioidosis affirm the significance of hot-spots in Australia and Thailand. However, it also highlights the paucity of systematic data from South Asia, The Americas, and Africa. Globally, the growing incidence of diabetes, chronic renal and (alcoholic) liver diseases further increase the susceptibility of individuals to *B. pseudomallei* infection. Recent outbreaks in nonendemic regions have exposed the hazard from the trade of animals and products as potential reservoirs for *B. pseudomallei*. Lastly, global warming will increase precipitation, severe weather events, soil salinity and anthrosol, all associated with the occurrence of *B. pseudomallei.*

**Summary:**

Epidemiological transitions, zoonotic hazards, and climate change are all contributing to the emergence of novel melioidosis-endemic areas. The adoption of the One Health approach involving multidisciplinary collaboration is important in unraveling the real incidence of *B. pseudomallei*, as well as reducing the spread and associated mortality.

## INTRODUCTION

Melioidosis, an infectious disease with reported case fatality rates between 10 and 50% [1], is caused by the Gram-negative soil bacterium *Burkholderia pseudomallei*[2]. The disease is endemic in Southeast Asia and Northern Australia, although more regions of endemicity are recently uncovered in Africa [3,4], South Asia [5], the Pacific, and the Americas [2,6]. Its impact on healthcare across the globe is significant. A recent report estimates the global burden of melioidosis in terms of disability-adjusted life years to be 4.6 million outperforming many other tropical diseases, such as dengue and schistosomiasis [7,8,9^▪^]. Surprisingly, however, melioidosis is still not officially listed as a neglected tropical disease by the WHO.

Infection occurs through skin penetration, direct contact with contaminated water or soil, ingestion of contaminated water, and inhalation of dust or water droplets [10]. Disease presentation ranges from acute sepsis or pneumonia to chronic illness with abscess formation. The incubation period of melioidosis ranges from 1 to 21 days, and reports of latency go up to 29 years [11]. Melioidosis is mainly considered an opportunistic disease as individuals with an altered immune function because of comorbidities – such as diabetes, renal, and liver failure – are at risk for infection and may have impaired disease outcomes [12^▪▪^].

**FIGURE 1:**
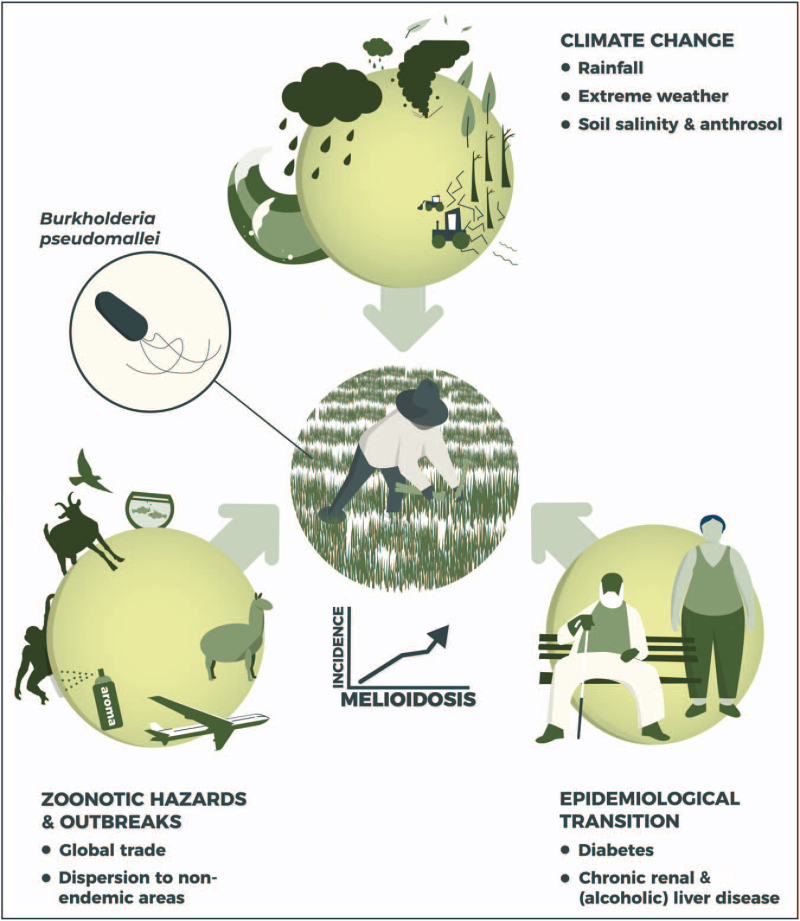
The drivers behind the increasing endemicity of melioidosis. Several factors can be identified that are likely to contribute to an increasing endemicity of melioidosis. Epidemiological transitions leading to a significant rise of comorbidities, such as diabetes, chronic kidney, and (alcoholic) liver failure, will continue to increase the number of individuals at risk for melioidosis. Zoonotic hazards, such as importation of exotic animals and livestock from endemic areas, may spread melioidosis to nonendemic regions. Climate change causes increased precipitation, severe weather events, and soil salinity and anthrosol, which may lead to expansion of the geographic dissemination and incidence of *Burkholderia pseudomallei*.

Human health has developed in unprecedented ways. As a consequence, individuals become older and suffer more frequently from comorbidities. Together with advances in mobility and globalization this might increase the susceptibility of individuals to emerging infectious diseases, such as melioidosis [13]. The global trade of animals and products, and global warming may further impact these changes. Here, we will review current insights into the increasing endemicity of melioidosis, focusing on epidemiological transitions, zoonotic hazards, and climate change (Fig. 1).

**Box 1:**
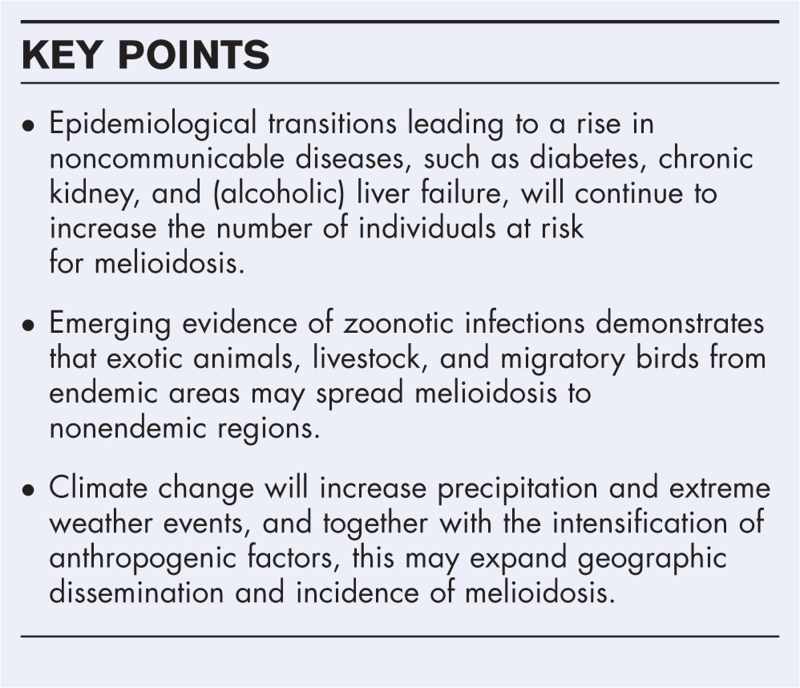
no caption available

## CURRENT INSIGHTS INTO THE EPIDEMIOLOGY OF MELIOIDOSIS

Current estimates suggest that melioidosis accounts globally for approximately 165 000 (68 000–412 000) cases and 89 000 (36 000–227 000) deaths per year [7]. *B. pseudomallei* is predominantly present in subtropical and tropical regions with Northern Australia and South-East Asia known as endemic hotspots. An overview on the reported phylogenomic reconstruction of the dissemination of *B. pseudomallei* across the globe is provided in the Conclusion section.

In Northern Australia, the annual melioidosis incidence is 4.8–51.2 (median 20.5) per 100 000 people [12^▪▪^]. The Darwin Prospective Melioidosis Study, which recently reported data from 1148 patients, has provided key insights into the clinical and microbiology characteristics of the disease [12^▪▪^]. Although melioidosis patients can present with a wide range of symptoms, more than half presents with pneumonia [14,15]. This extensive registry highlights the notion that *B. pseudomallei* is predominantly an opportunistic pathogen as most patients have clinical risk factors, such as diabetes and hazardous alcohol use. Individuals with one or multiple risk factors are more than eight times as likely to die from melioidosis. Moreover, the absence of risk factors is strongly correlated with survival as only 3 of 186 patients without risk factors died from melioidosis [14,15]. Furthermore, the study indicates a relatively low proportion of melioidosis in children but a greater number of infections in indigenous Australians, explained by high comorbidity rates and environmental exposure to the pathogen [12^▪▪^]. The authors state that early diagnosis, availability of appropriate antimicrobial therapy, and state-of-the-art intensive care therapy, could reduce mortality rates to less than 10% [12^▪▪^].

The South-East Asian WHO region covers more than 60% of the estimated global burden, with the highest total burden in India, with 1.6 million DALYs [7,8]. A recent cohort study described the clinical manifestations and outcomes of 114 individuals with *B. pseudomallei* infection presented in a tertiary hospital in southern India [16]. More than 80% of individuals had diabetes, which is more than globally reported and a probable reflection of the emerging diabetes epidemic in India [8,12^▪▪^,^16^,^17^]. A large clinical epidemiology study in Thailand determined 7126 culture-confirmed melioidosis cases from 2012 to 2015 across the country [18]. The average calculated incidence was 4 per 100 000 and was highest in Northeast Thailand (8.7 per 100 000) [18]. Geographical regions within Thailand showed a variance in age, comorbidities, and disease presentation, possibly because of differences in the environmental distribution of *B. pseudomallei*, in occupation, and in risk factors of affected patients. The overall 30-day case fatality rate in this cohort was 39%. However, only 4% of deaths were reported to Thailand's national notifiable disease surveillance system [18]. This study shows that integrating information from available data sets will improve national statistics and could support priority setting for policymakers.

Currently, Brazil accounts for over half of reported melioidosis cases in South America [19]. The state of Ceará in northeastern Brazil, has the highest reported incidence in South America and is the only region on the continent where specific infection prevention and control measurements have been implemented [19]. A retrospective study in this region on childhood melioidosis reported a mortality rate of 45% in 20 children. Most children presented with sepsis, pneumonia, and/or septic shock, although underreporting of mild cases could be a possible explanation for these severe outcomes [20^▪^]. Growing awareness and improvement of diagnostic facilities could lead to increased recognition of melioidosis in areas where the disease was previously absent [2]. A recent retrospective survey highlights this finding and identified 12 cases in Panama between 2007 and 2017 [21^▪^]. Most patients came from rural areas, and all occurred during the rainy season. Two patients were previously misidentified as tuberculosis as most clinical symptoms of melioidosis are nonspecific [21^▪^].

*B. pseudomallei* is considered endemic in Africa with estimates suggesting the presence of large numbers of underreported infection across the continent [7,8]. Most reported cases are in travelers returning from the African continent, as exemplified by a recent case of melioidosis presenting as chronic femoral osteomyelitis in a Ghanaian man living in the United Kingdom [3,4,22^▪^]. A comprehensive Dutch retrospective surveillance study of 33 travelers with *B. pseudomallei* infection showed that imported melioidosis could additionally serve as sentinels for detecting the disease in areas not yet considered melioidosis-endemic, such as The Gambia [23]. A recent single surveillance study demonstrated a relatively low estimated incidence of 1.3–2 per 1 million person-years using blood cultures in Kilify Country, Kenya [24]. These incidence rates might be an underestimation since approximately 50–75% [2] of melioidosis patients present with bacteremia, and culture has only an estimated sensitivity of around 60% [25]. Additionally, *B. pseudomallei* is commonly misidentified by biochemical identification systems or remains unidentified, even in endemic regions [26].

## EPIDEMIOLOGICAL TRANSITION

Epidemiological transition refers to changing population patterns in relation to life expectancy, mortality, fertility, and leading causes of death [27]. Accelerated mobility, developments in medical technology, and globalization have advanced the medical field in unprecedented ways. The world's population is ageing and individuals frequently suffer from comorbidities. These developments could make individuals more vulnerable to emerging infectious diseases, such as melioidosis. Given the rise in the numbers of immunocompromised travelers the incidence of imported melioidosis might also increase [23]. Most importantly, current epidemiological transitions in low-income and middle-income countries (LMICs) may lead to increased rates of individuals with risk factors for melioidosis.

Diabetes is present in 39–80% of cases depending on the cohort studied. Notably, people living with diabetes are 12 times as likely to get infected with *B. pseudomallei*[12^▪▪^,^16^,^28^,^29^]. As a comparison, in tuberculosis the presence of diabetes constitutes a three-fold increased risk to acquire the disease [30]. Numerous immunological studies demonstrate that diabetic patients have a reduced phagocytosis and killing capacity of *B. pseudomallei* as well as marked alterations in neutrophilic and monocytic cytokine responses [31–35]. Additionally, profound hyperglycemia is associated with higher infection rates and mortality because of bacterial infections [36]. Overall, the number of undiagnosed or poorly regulated diabetes patients in LMICs significantly exceeds numbers in high-income countries [35–37]. In 20 years, the total number of patients living with diabetes is estimated to increase by 55%, with more than 80% of diabetic patients living in LMICs [36].

Approximately 9–50% of melioidosis cases are reported to have underlying renal disease depending on the study location [8,12^▪▪^,^38^]. As with diabetes, chronic kidney disease is associated with impaired chemotaxis and reduced phagocytic capacity as demonstrated by experimental studies on other pathogens [39,40]. In 2017, the number of people receiving renal replacement therapy was estimated to be around 2.5 million worldwide, and this number will most likely have doubled in 2030 [41^▪^].

The earlier reported Darwin study describes hazardous alcohol use in 39% of cases, predominantly in disadvantaged communities [42]. Globally, chronic liver disease is present in approximately 7% of patients, according to a systematic review on the global burden of melioidosis [8]. Observed immune alterations in alcohol-related liver disease have overlapped with those seen in individuals with diabetes and renal impairment [43]. An experimental study showed that binge drinking impaired innate immune functions through increased barrier permeability during *B. pseudomallei* infection [44]. Notably, around 20–30% of mortality attributed to cirrhosis and liver cancer in the Asia-Pacific region are attributable to hazardous alcohol consumption [45,46^▪^]. Moreover, the prevalence of nonalcohol fatty liver disease will rise because of obesity and diabetes [47,48^▪^].

In short, epidemiological transitions further increase the susceptibility of individuals to *B. pseudomallei* infection in melioidosis-endemic regions (Fig. 1) [41^▪^]. LMICs will carry a double burden of disease with potential increasing numbers of both noncommunicable diseases and emerging infectious diseases like melioidosis [35,36].

## OUTBREAKS AND ZOONOTIC HAZARDS

Although melioidosis is not always considered a zoonotic disease, animals can shed *B. pseudomallei* in the environment [49^▪^]. A wide range of animal species are susceptible for infection with *B. pseudomallei,* such as goats, sheep, camels, and alpacas [50]. Crocodiles, previously known as being highly resistant to the disease, also demonstrated susceptibility to the bacterium. A cluster of melioidosis infections in saltwater crocodiles of which two were fatal was recently described in Northern Australia [51]. Interestingly, several case studies reported that imported exotic animals, nonhuman primates, pet iguanas, and a canine transmitted *B. pseudomallei* to nonendemic regions, among others to the United States and Europe [52–55]. Although rare, infection outbreaks have been reported in temperate climatic regions and are generally attributable to a single origin in the environment. For example, several outbreaks have been reported in piggeries in Queensland, Australia [56], in primates imported from the Philippines to the United Kingdom [57], and in an donated panda from China to a zoo in Paris, France [58]. Additionally, following high rainfall, a melioidosis cluster in 23 alpacas (*Vicugna pacos*) and a parrot (*Ara macao*) was reported in southwest Australia [59^▪^]. More than 20 animals died on the alpaca farm. Genome sequencing indicated that *B. pseudomallei* can survive in nontropical environments in a latent state and activate later following favorable conditions [59^▪^]. Another example of possible zoonotic transmission is a recent report of a human case contracting melioidosis from her tropical fish aquarium in the United States [60^▪▪^]. Genetic analysis revealed clonal matches between the human isolate and those from her freshwater home aquarium [60^▪▪^]. A case of aquarium water contaminated by an imported tropical fish from Singapore has been earlier reported in France [61]. Overall, animals should be seriously considered as potential hazards in transmitting melioidosis to humans (Fig. 1).

Most melioidosis cases reported in the United States are associated with travel to endemic areas. However, sporadic cases with no travel history suggest possible domestic exposure, for example, to contaminated products [6,62–64]. In some cases, genetic investigation of *B. pseudomallei* strains from human cases pointed towards South and East Asia as their probable origin. Recently, the CDC confirmed a multistate outbreak of melioidosis in four patients living in Georgia, Kansas, Minnesota, and Texas [65^▪▪^]. All four cases had no travel history to melioidosis-endemic areas. Only two of them were adults with comorbidities recognized as risk factors for melioidosis. The other two were children without a relevant medical history. Genetic analysis on the isolates indicated a genetic origin closely related to South Asia, suggesting a common source of infection. An extensive public health investigation on several environmental and consumer products revealed an aromatherapy spray as the source of infection in the Georgia's patient's house. The genetic fingerprint of the isolated strain genetically matched the *B. pseudomallei* strains in all four patients. This finding illustrates that the aromatherapy spray (or a component) was the source of infection in all four cases [65^▪▪^]. Earlier reports of irrigation fluids and hand detergents resulting in clusters of cutaneous melioidosis indicated the danger of contaminated products [66]. Therefore, *B. pseudomallei* infection should also be considered in the differential diagnosis in patients that matches the characteristics of melioidosis even in the absence of a travel history to endemic areas. Whole-genome sequencing can result in epidemiological insights and can provide knowledge about the transmission and environmental dispersal patterns.

## CLIMATE CHANGE

Climate change could directly impact on the distribution of *B. pseudomallei* and the seasonal pattern of the disease. It is anticipated that global temperatures will continue to rise by 0.2 °C per decade even if greenhouse gas emissions are reduced drastically in the coming decades [67^▪▪^,^68^^▪▪^]. This ongoing temperature rise will increase precipitation, extreme weather events, and droughts [67^▪▪^,^68^^▪▪^,^69^]. Precipitation will decrease in some parts of the subtropics and tropics, while particularly the high latitudes, equatorial Pacific, and some monsoon regions will experience a heavy increase in the format of intense tropical storms [67^▪▪^,^68^^▪▪^]. Monsoon rainfall is expected to increase, particularly in South-Asia, East-Asia, South-East Asia, and West Africa [67^▪▪^,^68^^▪▪^].

For a long time, it has been recognized that rainfall leads to increased *B. pseudomallei* occurrence in the soil [70]. This correlation is most robust in melioidosis endemic hotspots, such as South-East Asia and Northern Australia, possibly because of the extreme precipitation experienced during the monsoon season. For instance, studies in Singapore and Malaysia indicated that the number of cases admitted to the hospital was directly related to the severity of rainfall and that melioidosis as such should be considered a seasonal disease [71,72]. The Darwin study demonstrated that for every 100 mm of rainfall, melioidosis incidence increased by 14% in Northern Australia [12^▪▪^]. In line, a study conducted in Laos and Cambodia showed that humidity and windy conditions significantly influence the seasonal burden of melioidosis [73]. During such periods of very high humidity, children appeared to have a three times higher risk of infection with *B. pseudomallei* compared with adults. Windy conditions were associated with pulmonary and disseminated infections [73]. Additionally, it has been shown that rivers can serve as potential carriers for *B. pseudomallei* and can facilitate the spread of the pathogen over long distances to nonendemic melioidosis areas [74].

Extreme weather events are frequently linked to higher melioidosis incidence rates. This is illustrated by the occurrence of clusters of *B. pseudomallei* infection after a typhoon and subsequent widespread flooding in Taiwan [75,76], a tsunami in Thailand [77], and tropical cyclones in Northern Australia [78]. The high-velocity wind that accompanies such events could lead to inhalation of aerosolized *B. pseudomallei* as indicated by earlier studies [75,79]. Recently, heavy rainfall and flooding in Sri Lanka caused a case cluster of 10 patients [80^▪▪^]. Moreover, extreme weather events can also activate *B. pseudomallei* in regions where the bacterium is latently present in the environment as has been demonstrated in central Australia [81]. The chances of flooding increase with sea-rise and intensification of anthropogenic activities in coastal areas, such as urban development, fisheries, and agricultural practices. As a consequence, protective ecosystems like mangroves and coral reefs are frequently lost. Subsequently, flooding can lead to higher salinization and anthrosol soil [7,82,83]. A prediction model found that high rainfall, soil types (e.g. anthrosol and acrisol), and salinity were all strongly correlated with the presence of *B. pseudomallei* in the environment [7]. Therefore, these factors were used to estimate the incidence of melioidosis globally. Of note, the model predicted that multiple regions suitable *for B. pseudomallei* establishment are present in Japan and the United States (e.g. Florida, Louisiana, and Texas) [7].

Continued global warming will also affect global migration patterns [82,84^▪^,^85^,^86^]. Food uncertainty because of heatwaves and droughts can become more frequent [82]. This may instigate the movement of people and agricultural practices from arid areas to more fertile and wet regions, consequently leading to a rise in the total number of at risk individuals living in melioidosis endemic areas [82,85]. Growing agricultural practices in these regions will increase anthrosol, promoting *B. pseudomallei* suitability. Overall, human-induced climate change increases favorable conditions for *B. pseudomallei* in and outside endemic areas worldwide [67^▪▪^,^68^^▪▪^] (Fig. 1).

## THE ROAD AHEAD

As illustrated above, the epidemiology of melioidosis will be impacted by human, animal, and environmental developments. As a consequence, it is advisable to adopt the One Health approach as an integrated view recognizing this triad interconnection [87]. The One Health approach aims at better public health outcomes through monitoring and surveillance, and will be crucial in determining the actual incidence of *B. pseudomallei* in humans, animals, and the environment. Interdisciplinary, multidisciplinary, and transdisciplinary collaborations between human health, animal health, and environment sectors should be strengthened on a local, national, and global level to address the spread of melioidosis and decrease patient vulnerability [88]. By implementing the One Health approach, best practices can be exchanged by educating and engaging local communities, implementing preventive behavioral programs [89], and improving laboratories in resource-limited areas [2]. We recommended to use the current interactive tools and materials at www.melioidosis.info. Further, cooperating of the clinical field and basic scientists with veterinarians, ecologists, environmental experts, and agricultural professionals could bring innovative solutions to impact indirect drivers of melioidosis. These actions must take place not only in endemic areas but also in nonendemic susceptible regions.

## CONCLUSION

Estimates of the global burden of melioidosis not only affirm the significance of hot-spots in Australia and Thailand but also highlight the paucity of systematic data from South Asia, the Americas, and Africa. Many LMIC countries are going through an epidemiological transition, leading to a significant rise in individuals with risk factors of melioidosis, such as diabetes, chronic kidney, and (alcoholic) liver failure. Recent outbreaks of melioidosis in nonendemic regions demonstrate that zoonotic infections combined with global trade and animal migratory patterns can cause the dispersion of *B. pseudomallei* to nonendemic areas. Moreover, given that *B. pseudomallei* is a soil-dwelling bacterium that causes a seasonal disease, climate change will impact the melioidosis’ incidence and geographic dissemination. Melioidosis is often challenging to diagnose because of a wide range of disease presentations, low awareness, and a lack of good laboratory facilities. There is a need for better granularity of data on the worldwide distribution of *B. pseudomallei* to prevent and decrease mortality and morbidity from this disease. The adoption of the One Health approach involving multidisciplinary collaboration in all aspects of healthcare for humans, animals, and the environment could help to determine the real incidence and decrease the spread and morbidity associated with *B. pseudomallei* infection globally.

### Phylogenomic reconstruction of the dissemination of *B. pseudomallei* across the globe; a genetic window to the past

The first case of melioidosis was identified in Myanmar in 1911 [90]. Chewapreecha *et al.*[91] Showed that *B. pseudomallei* most likely originated in Australia. By using whole-genome sequencing on 500 bacterial isolates from 30 countries, the authors could demonstrate that the movement of people and products has led to the dissemination of *B. pseudomallei* across the globe [91]. Approximately 16 000–225 000 years ago, during the last glacial period, *B. pseudomallei* was transferred from Australia to Southeast and East Asia [91,92]. Subsequently, historical trade routes, migration of populations, and migratory birds spread the bacterium throughout Asia [93–95]. For example, a study conducted in the melioidosis-endemic Darwin region in Australia identified *B. pseudomallei* in the beaks of wild birds, such as native finches and doves [93].

Melioidosis has probably been introduced in Madagascar 1500–2000 years ago. Phylogenomic reconstruction demonstrated that African isolates grouped together with Asian strains, indicating Asian origins [91,96]. The introduction of pigs, a known reservoir of melioidosis, and migratory birds could be a possible explanation of the dissemination of *B. pseudomallei* from Asia to Madagascar [97]. Next, anthropogenic factors, like human migration and trade routes, probably dispersed the disease to the African mainland [96]. Cargos transported during the transatlantic slave trade, such as contaminated food, water, plants, and animals, could have introduced *B. pseudomallei* into the Americas between 1650 and 1850. Strong genetic similarities between isolates from Africa and America supported this finding [96].

## Acknowledgements

*We would like to thank many members of the worldwide melioidosis community, embodied in the International Melioidosis Network, for all the insightful discussions over the last years leading to this work.*

### Financial support and sponsorship

*This work was supported by a Research Grant (2018) from the European Society of Clinical Microbiology and Infectious Diseases (ESCMID to E.B.) and the Netherlands Organization for Scientific Research (VIDI grant number 91716475 to W.J.W.).*

### Conflicts of interest

*There are no conflicts of interest.*

## REFERENCES AND RECOMMENDED READING

Papers of particular interest, published within the annual period of review, have been highlighted as:

▪ of special interest
▪▪ of outstanding interest
